# Editorial: Pathogenesis, Clinical Findings, and Treatment Advances in Kawasaki Disease

**DOI:** 10.3389/fped.2021.781842

**Published:** 2021-10-25

**Authors:** Teresa Giani, Isabelle Koné-Paut, Rolando Cimaz, Caroline Galeotti

**Affiliations:** ^1^Department of Medical Biotechnology, University of Siena, Siena, Italy; ^2^Department of Pediatric Rheumatology and CEREMAIA, University of Paris Saclay, Bicêtre Hospital, APHP, Paris, France; ^3^Department of Clinical Sciences and Community Health, University of Milan, Milan, Italy

**Keywords:** Kawasaki, coronary, coronary arterial lesions, aneurysm, vasculitis

The recent Research Topic on Kawasaki disease (KD) covers some interesting issues; some have been dealt with during the first European meeting on KD, which was held in Paris in January 2021 and which was organized by two of the authors (RC and IKP). The topic includes 11 articles, and covers epidemiology (*n* = one article), etiopathogenesis (*n* = 2), clinical aspects (*n* = 3), imaging (*n* = 1), treatment (*n* = 2), and the “hot” subject of COVID19-related multisystem inflammatory syndrome in children (MIS-C) (*n* = 2).

The epidemiology of KD varies throughout the world. The disease is well-known to be more frequent in Asia, with figures more than 20-fold higher in Japanese when compared to Caucasians. Piram in her article showed that the annual incidence of KD in northern and western European countries is about 10–15 per 100,000 children under 5 years of age and seems to be relatively stable over time. From the limited data available, the incidence seems to be similar in Eastern and Western Europe. Demographic characteristics of KD in Europe are in line with those in other countries.

Although the exact pathogenesis of KD is still unclear, possible triggers may be viral infections, which remain most often unidentified. According to a study, systemic immune responses early in life could protect against developing KD, so the incidence of previous Cytomegalovirus (CMV) or Epstein-Barr virus (EBV) infections was investigated in children with KD compared to healthy age-matched controls (van Stijn et al.). By comparing 86 KD patients with an age-matched group with regard to CMV and EBV VCA IgG measurements (taken before or 9 months after IVIG treatment), the authors found that both CMV and EBV had an almost 2-fold lower seroprevalence in the KD population than in the control group.

The group of Arditi in Los Angeles provided an interesting paper on an animal model (Maruyama et al.). miRNAs inhibit mRNA translation or decrease the levels of their corresponding mRNA, thereby inhibiting targeted protein synthesis. miR-223 has been shown to be up-regulated in the blood and coronary tissue of KD patients. This study shows that miR-223 expression is up-regulated in inflamed aneurysms and dilatations of LCWE-injected mice, and that mice lacking miR-223 have more vessel inflammation and IL-1 beta secretion. Thus, the authors have hypothesized that intense IL-1 beta production during LCWE-induced KD vasculitis induces miR-223 expression as a negative regulatory feedback loop.

Concerning clinical aspects, the criteria that we still rely on are those endorsed by AHA and AAP (1). However, in many cases the diagnosis might be difficult, since KD may mimic viral infections and other inflammatory disorders such as systemic onset JIA (sJIA). Go et al. suggested that sJIA and KD might belong to the same spectrum of IL-1 mediated cytokine storm syndromes. They evaluated in a large retrospective study of patients with KD (*n* = 1,765) and sJIA (*n* = 112) the frequency of patients combining the two diagnoses (KD/sJIA). Seven percent (75% males) fulfilled both criteria; they had a median age of 4.7 years, hence higher than that of KD patients. KD/sJIA had less conjunctival injection and half had incomplete KD features. They also received more than one IVIg infusion and 62.5% had coronary dilatation. In these patients, arthritis appeared between 1 and 4 months after the onset of symptoms.

Of note, arthritis is a complication of KD that occurs in up to one third of untreated patients. In order to stratify patients for risk factors and therefore for treatment selection during the course of KD, a nationwide survey in Japan was conducted on patients who developed arthritis requiring a treatment (Kanemasa et al.). The serum levels of ferritin and IL-18 or the combination of clinical covariates at the onset of arthritis could predict the treatment response and prognosis of arthritis.

Being a systemic vasculitis KD can affect any organ system. However, neurologic involvement is often underdiagnosed. Maggio et al., showed that abnormalities of brainstem auditory evoked potentials were associated with the risk of coronary artery lesions. The authors suggested that this might be a concomitant subclinical vasculitis of *vasa nervorum*.

However, the more dreadful complication is undoubtedly coronary involvement; van Stijn et al., in their retrospective single-center study compared the diagnostic yield of coronary computed tomographic angiography and cardiac magnetic resonance imaging (MRI) for the detection of coronary abnormalities. Coronary computed tomographic angiography could assess the coronary artery tree at great resolution, identifying coronary artery aneurysms more frequently and with greater detail when compared to cardiac MRI.

We cannot ignore in this pandemic time the subject of MIS-C (2, 3); indeed, two articles tried to analyze the differences and similarities between this entity and KD. A Spanish study has compared the KD cases diagnosed according to the AHA criteria during the COVID-19 period (March–May, 2020) that were either SARS-CoV-2 confirmed (CoV+) or negative (CoV–) by PCR and serology, to those from the same period during 2018 and 2019 (PreCoV) (Fernández-Cooke et al.). The CoV+ group included a significantly higher proportion of non-Caucasians (64%) than Caucasians (25%). Cases and intensive care admissions increased significantly in 2020 during the CoVID-19 period (CoV−20% and CoV+ 50%, *P* < 0.001, vs. 1.6% of patients during the PreCoV).

Moreover, a Japanese observational study aimed to identify similarities and differences between Kawasaki Disease Shock Syndrome (KDSS) and MIS-C (Suzuki et al.). KDSS was more likely to have a diagnosis of complete KD, a higher incidence of coronary artery abnormalities (CAAs) (50 vs. 11%) and a greater requirement for vasoactive agonists. 6.7% of patients with KDSS died and 1.7% of patients with MIS-C died.

Finally, treatment issues have also been dealt with, in particular regarding the use of intravenous immunoglobulins (IVIG). In a Japanese study, a machine learning method used commonly available clinical and laboratory variables, in order to predict resistance to IVIG therapy (Kuniyoshi et al.). Three models based only on demographics and routine laboratory variables did not provide reliable performances. Since the identification of high-risk patients is crucial for early aggressive treatment, we still need further research including the addition of biomarkers to the model.

Lastly, in a systematic review and meta-analysis investigating the effects of early IVIG therapy (administration <5 days from disease onset), 14 studies involving 70,396 patients were included (Yan et al.). Early treatment with IVIG can lead to an increased risk of IVIG unresponsiveness [OR 2.24; 95% CI (1.76, 2.84); *p* < 0.001]. However, in contrast to studies performed in Japan that found no significant difference in coronary artery lesions (CAL) development, studies conducted in China and in the United States showed a reduced risk in the occurrence of CAL with early IVIG treatment.

## Author Contributions

All authors listed have made a substantial, direct and intellectual contribution to the work, and approved it for publication.

## Conflict of Interest

The authors declare that the research was conducted in the absence of any commercial or financial relationships that could be construed as a potential conflict of interest.

## Publisher's Note

All claims expressed in this article are solely those of the authors and do not necessarily represent those of their affiliated organizations, or those of the publisher, the editors and the reviewers. Any product that may be evaluated in this article, or claim that may be made by its manufacturer, is not guaranteed or endorsed by the publisher.
